# Supplementary material for: Meta-Analysis and Gene Set Enrichment Relative to ER Status Reveal Elevated Activity of MYC and E2F in the “Basal” Breast Cancer Subgroup
Source: PLoS One. 2009 Mar 9;4(3):e4710. doi: 10.1371/journal.pone.0004710 (PMC2650420; doi:10.1371/journal.pone.0004710)
Supplement: Dataset S1 — GSEA results from first screen. The file contains a zipped archive of the HTML results of the second GSEA screen. Reports for enriched or depleted datasets may be accessed from the “index.html” link within the my_analysis.GseaPreranked.1219975950625.rpt.zip ZIP archive. Enrichment plots are available for each of the 50 most enriched and depleted gene sets. (13.32 MB ZIP) [file pone.0004710.s001.zip › my_analysis.GseaPreranked.1219975950625.rpt.zip!//LI_FETAL_VS_WT_KIDNEY_DN.html]

Details for gene set LI\_FETAL\_VS\_WT\_KIDNEY\_DN[GSEA]

|  || Dataset | zSco.ERneg.to.ERpos\_collapsed |
| Phenotype | NoPhenotypeAvailable |
| Upregulated in class | na\_pos |
| GeneSet | LI\_FETAL\_VS\_WT\_KIDNEY\_DN |
| Enrichment Score (ES) | 0.56134427 |
| Normalized Enrichment Score (NES) | 2.460911 |
| Nominal p-value | 0.0 |
| FDR q-value | 0.0 |
| FWER p-Value | 0.0 |
Table: GSEA Results Summary

  

Fig 1: Enrichment plot: LI\_FETAL\_VS\_WT\_KIDNEY\_DN      
 Profile of the Running ES Score & Positions of GeneSet Members on the Rank Ordered List

  

| PROBE | GENE SYMBOL | GENE\_TITLE | RANK IN GENE LIST | RANK METRIC SCORE | RUNNING ES | CORE ENRICHMENT || 1 | CDC20 |  |  | 9 | 7.151 | 0.0144 | Yes |
| 2 | TTK |  |  | 11 | 7.059 | 0.0292 | Yes |
| 3 | SRPK1 |  |  | 12 | 6.990 | 0.0439 | Yes |
| 4 | TMSL8 |  |  | 50 | 6.319 | 0.0544 | Yes |
| 5 | BLM |  |  | 56 | 6.255 | 0.0672 | Yes |
| 6 | NASP |  |  | 59 | 6.201 | 0.0801 | Yes |
| 7 | AURKB |  |  | 76 | 6.038 | 0.0916 | Yes |
| 8 | TAF5 |  |  | 81 | 6.010 | 0.1040 | Yes |
| 9 | MLXIP |  |  | 88 | 5.965 | 0.1161 | Yes |
| 10 | TRIP13 |  |  | 96 | 5.890 | 0.1280 | Yes |
| 11 | MKI67 |  |  | 98 | 5.871 | 0.1403 | Yes |
| 12 | MAP4K4 |  |  | 115 | 5.747 | 0.1512 | Yes |
| 13 | KIF2C |  |  | 124 | 5.703 | 0.1626 | Yes |
| 14 | UCK2 |  |  | 132 | 5.685 | 0.1740 | Yes |
| 15 | KIFC1 |  |  | 156 | 5.525 | 0.1839 | Yes |
| 16 | MELK |  |  | 181 | 5.424 | 0.1935 | Yes |
| 17 | MCM6 |  |  | 198 | 5.317 | 0.2035 | Yes |
| 18 | MTHFD2 |  |  | 205 | 5.307 | 0.2142 | Yes |
| 19 | KIF14 |  |  | 221 | 5.215 | 0.2241 | Yes |
| 20 | PTTG1 |  |  | 225 | 5.206 | 0.2348 | Yes |
| 21 | TPX2 |  |  | 231 | 5.180 | 0.2453 | Yes |
| 22 | CKS2 |  |  | 239 | 5.145 | 0.2556 | Yes |
| 23 | TP53BP2 |  |  | 245 | 5.119 | 0.2660 | Yes |
| 24 | CENPF |  |  | 268 | 5.028 | 0.2750 | Yes |
| 25 | BUB1B |  |  | 271 | 5.016 | 0.2854 | Yes |
| 26 | SHMT2 |  |  | 308 | 4.920 | 0.2930 | Yes |
| 27 | STMN1 |  |  | 310 | 4.907 | 0.3033 | Yes |
| 28 | EZH2 |  |  | 312 | 4.888 | 0.3135 | Yes |
| 29 | CKS1B |  |  | 416 | 4.638 | 0.3154 | Yes |
| 30 | CCNB2 |  |  | 431 | 4.589 | 0.3240 | Yes |
| 31 | SMC4 |  |  | 448 | 4.519 | 0.3323 | Yes |
| 32 | NUP205 |  |  | 459 | 4.484 | 0.3410 | Yes |
| 33 | AURKA |  |  | 460 | 4.483 | 0.3505 | Yes |
| 34 | MAD2L1 |  |  | 477 | 4.446 | 0.3586 | Yes |
| 35 | DLG7 |  |  | 521 | 4.306 | 0.3644 | Yes |
| 36 | PLK4 |  |  | 524 | 4.292 | 0.3733 | Yes |
| 37 | ASNS |  |  | 618 | 4.099 | 0.3749 | Yes |
| 38 | MCM2 |  |  | 630 | 4.080 | 0.3827 | Yes |
| 39 | SLC16A1 |  |  | 638 | 4.055 | 0.3907 | Yes |
| 40 | ST6GAL1 |  |  | 649 | 4.034 | 0.3984 | Yes |
| 41 | WASF3 |  |  | 686 | 3.963 | 0.4040 | Yes |
| 42 | APOBEC3B |  |  | 701 | 3.929 | 0.4112 | Yes |
| 43 | KIF11 |  |  | 713 | 3.909 | 0.4186 | Yes |
| 44 | FEN1 |  |  | 754 | 3.838 | 0.4237 | Yes |
| 45 | FZD7 |  |  | 767 | 3.811 | 0.4308 | Yes |
| 46 | MSH2 |  |  | 819 | 3.748 | 0.4348 | Yes |
| 47 | DKC1 |  |  | 924 | 3.607 | 0.4345 | Yes |
| 48 | PRIM2A |  |  | 941 | 3.564 | 0.4408 | Yes |
| 49 | CHAF1A |  |  | 971 | 3.525 | 0.4460 | Yes |
| 50 | IARS |  |  | 987 | 3.501 | 0.4523 | Yes |
| 51 | BOP1 /// LOC653119 |  |  | 1001 | 3.482 | 0.4586 | Yes |
| 52 | KIF23 |  |  | 1030 | 3.442 | 0.4637 | Yes |
| 53 | SACS |  |  | 1039 | 3.426 | 0.4703 | Yes |
| 54 | SNRPA1 |  |  | 1064 | 3.399 | 0.4757 | Yes |
| 55 | UNG |  |  | 1070 | 3.390 | 0.4824 | Yes |
| 56 | NUP188 |  |  | 1097 | 3.364 | 0.4876 | Yes |
| 57 | PFKP |  |  | 1165 | 3.281 | 0.4894 | Yes |
| 58 | YARS |  |  | 1177 | 3.262 | 0.4954 | Yes |
| 59 | C12ORF24 |  |  | 1215 | 3.220 | 0.4994 | Yes |
| 60 | NOLC1 |  |  | 1222 | 3.217 | 0.5057 | Yes |
| 61 | DNAJC9 |  |  | 1243 | 3.198 | 0.5109 | Yes |
| 62 | CDC2 |  |  | 1259 | 3.182 | 0.5165 | Yes |
| 63 | MSH6 |  |  | 1276 | 3.164 | 0.5219 | Yes |
| 64 | GTF3C2 |  |  | 1339 | 3.094 | 0.5238 | Yes |
| 65 | PCNA |  |  | 1346 | 3.087 | 0.5298 | Yes |
| 66 | CIT |  |  | 1464 | 2.983 | 0.5272 | Yes |
| 67 | XPOT |  |  | 1517 | 2.930 | 0.5294 | Yes |
| 68 | SSRP1 |  |  | 1591 | 2.860 | 0.5299 | Yes |
| 69 | NCBP2 |  |  | 1601 | 2.850 | 0.5352 | Yes |
| 70 | ILF2 |  |  | 1644 | 2.807 | 0.5379 | Yes |
| 71 | SEPT6 |  |  | 1656 | 2.796 | 0.5430 | Yes |
| 72 | KIAA1794 |  |  | 1724 | 2.747 | 0.5437 | Yes |
| 73 | CKAP5 |  |  | 1738 | 2.731 | 0.5485 | Yes |
| 74 | BAZ1B |  |  | 1742 | 2.730 | 0.5540 | Yes |
| 75 | CDC25C |  |  | 1794 | 2.700 | 0.5558 | Yes |
| 76 | CDKN3 |  |  | 1827 | 2.674 | 0.5590 | Yes |
| 77 | MIZF |  |  | 1870 | 2.631 | 0.5613 | Yes |
| 78 | HMGA2 |  |  | 2058 | 2.478 | 0.5524 | No |
| 79 | CCT3 |  |  | 2071 | 2.470 | 0.5566 | No |
| 80 | H2AFZ |  |  | 2105 | 2.447 | 0.5593 | No |
| 81 | ACP1 |  |  | 2358 | 2.283 | 0.5450 | No |
| 82 | TIA1 |  |  | 2434 | 2.233 | 0.5440 | No |
| 83 | CCND2 |  |  | 2506 | 2.197 | 0.5432 | No |
| 84 | PARP1 |  |  | 2571 | 2.161 | 0.5429 | No |
| 85 | TXNRD1 |  |  | 2591 | 2.152 | 0.5460 | No |
| 86 | CDK2 |  |  | 2688 | 2.099 | 0.5431 | No |
| 87 | DDX1 |  |  | 2722 | 2.079 | 0.5450 | No |
| 88 | BAZ1A |  |  | 2724 | 2.078 | 0.5493 | No |
| 89 | HOXA9 |  |  | 2762 | 2.062 | 0.5508 | No |
| 90 | LMNB1 |  |  | 2764 | 2.060 | 0.5551 | No |
| 91 | FADS1 |  |  | 2837 | 2.021 | 0.5539 | No |
| 92 | COL13A1 |  |  | 2874 | 1.999 | 0.5553 | No |
| 93 | MARS |  |  | 2964 | 1.952 | 0.5527 | No |
| 94 | CXCR4 |  |  | 3062 | 1.902 | 0.5493 | No |
| 95 | RNASEH2A |  |  | 3126 | 1.876 | 0.5485 | No |
| 96 | STRAP |  |  | 3251 | 1.816 | 0.5429 | No |
| 97 | NR2C1 |  |  | 3255 | 1.815 | 0.5465 | No |
| 98 | ABCA2 |  |  | 3367 | 1.764 | 0.5418 | No |
| 99 | NAP1L1 |  |  | 3421 | 1.740 | 0.5414 | No |
| 100 | TOPBP1 |  |  | 3475 | 1.722 | 0.5410 | No |
| 101 | MYL6B |  |  | 3506 | 1.705 | 0.5423 | No |
| 102 | CHN1 |  |  | 3715 | 1.616 | 0.5299 | No |
| 103 | TOP2A |  |  | 3735 | 1.610 | 0.5319 | No |
| 104 | KIAA0692 |  |  | 3863 | 1.567 | 0.5255 | No |
| 105 | SS18 |  |  | 3872 | 1.565 | 0.5282 | No |
| 106 | LRP4 |  |  | 3964 | 1.526 | 0.5245 | No |
| 107 | RFC5 |  |  | 4034 | 1.500 | 0.5224 | No |
| 108 | BTAF1 |  |  | 4519 | 1.311 | 0.4884 | No |
| 109 | YWHAZ |  |  | 4701 | 1.253 | 0.4773 | No |
| 110 | KIAA0101 |  |  | 4770 | 1.230 | 0.4747 | No |
| 111 | BCL7A |  |  | 4819 | 1.217 | 0.4737 | No |
| 112 | GARS |  |  | 4828 | 1.211 | 0.4756 | No |
| 113 | RRM1 |  |  | 4872 | 1.197 | 0.4749 | No |
| 114 | EYA1 |  |  | 5027 | 1.143 | 0.4656 | No |
| 115 | KIAA0515 |  |  | 5110 | 1.113 | 0.4617 | No |
| 116 | COL2A1 |  |  | 5293 | 1.050 | 0.4501 | No |
| 117 | DDX52 |  |  | 5360 | 1.024 | 0.4472 | No |
| 118 | IGSF4 |  |  | 5403 | 1.010 | 0.4462 | No |
| 119 | CDH2 |  |  | 5630 | 0.937 | 0.4310 | No |
| 120 | TIMELESS |  |  | 5760 | 0.892 | 0.4230 | No |
| 121 | POLE3 |  |  | 5825 | 0.870 | 0.4200 | No |
| 122 | ESPL1 |  |  | 5874 | 0.853 | 0.4182 | No |
| 123 | SPAG5 |  |  | 6030 | 0.800 | 0.4081 | No |
| 124 | FADS2 |  |  | 6236 | 0.735 | 0.3940 | No |
| 125 | GREB1 |  |  | 6264 | 0.727 | 0.3935 | No |
| 126 | PRIM1 |  |  | 6525 | 0.642 | 0.3751 | No |
| 127 | TCERG1 |  |  | 7338 | 0.369 | 0.3142 | No |
| 128 | ZNF423 |  |  | 7629 | 0.259 | 0.2927 | No |
| 129 | NME1 |  |  | 7807 | 0.187 | 0.2797 | No |
| 130 | XRCC5 |  |  | 8169 | 0.051 | 0.2523 | No |
| 131 | FLJ11021 |  |  | 8179 | 0.045 | 0.2517 | No |
| 132 | SV2A |  |  | 8625 | -0.140 | 0.2182 | No |
| 133 | BRCA1 |  |  | 8777 | -0.199 | 0.2072 | No |
| 134 | PPP2R2A |  |  | 9185 | -0.396 | 0.1771 | No |
| 135 | YAF2 |  |  | 9328 | -0.472 | 0.1673 | No |
| 136 | LIG3 |  |  | 9367 | -0.492 | 0.1654 | No |
| 137 | BUB3 |  |  | 9557 | -0.594 | 0.1523 | No |
| 138 | SCRN1 |  |  | 9991 | -0.836 | 0.1212 | No |
| 139 | PBX3 |  |  | 10267 | -1.001 | 0.1024 | No |
| 140 | MPHOSPH9 |  |  | 10286 | -1.019 | 0.1032 | No |
| 141 | CHST1 |  |  | 10648 | -1.279 | 0.0785 | No |
| 142 | MEOX1 |  |  | 10820 | -1.404 | 0.0684 | No |
| 143 | GCN1L1 |  |  | 10870 | -1.440 | 0.0677 | No |
| 144 | SIX1 |  |  | 11441 | -1.979 | 0.0286 | No |
| 145 | PCBP2 |  |  | 11508 | -2.045 | 0.0279 | No |
| 146 | CACNB3 |  |  | 11624 | -2.146 | 0.0237 | No |
| 147 | ROR2 |  |  | 11725 | -2.247 | 0.0208 | No |
| 148 | GPC1 |  |  | 11965 | -2.518 | 0.0080 | No |
| 149 | ADIPOR2 |  |  | 12026 | -2.591 | 0.0089 | No |
| 150 | NELL2 |  |  | 12593 | -3.420 | -0.0269 | No |
| 151 | MN1 |  |  | 12609 | -3.449 | -0.0208 | No |
| 152 | ELOVL2 |  |  | 12695 | -3.604 | -0.0197 | No |
| 153 | SALL2 |  |  | 12781 | -3.823 | -0.0181 | No |
| 154 | FAM62A |  |  | 12824 | -3.940 | -0.0130 | No |
| 155 | DLG5 |  |  | 12901 | -4.157 | -0.0100 | No |
| 156 | DDX23 |  |  | 13079 | -4.749 | -0.0134 | No |
| 157 | TARBP2 |  |  | 13087 | -4.776 | -0.0039 | No |
| 158 | CRABP2 |  |  | 13116 | -4.890 | 0.0043 | No |
| 159 | ZNF516 |  |  | 13186 | -5.288 | 0.0102 | No |
Table: GSEA details [plain text format]

  

Fig 2: LI\_FETAL\_VS\_WT\_KIDNEY\_DN: Random ES distribution      
 Gene set null distribution of ES for **LI\_FETAL\_VS\_WT\_KIDNEY\_DN**
